# Supplementary material for: Family imprint reveals basin-wide patterns of Amazon forest embolism resistance
Source: Nat Commun. 2026 Feb 26;17:2073. doi: 10.1038/s41467-026-69892-1 (PMC12949053; doi:10.1038/s41467-026-69892-1)
Supplement: Supplementary file 1 — Supplementary Information [file 41467_2026_69892_MOESM1_ESM.pdf]

Supplementary Information

Family imprint reveals basin-wide patterns of Amazon forest embolism resistance

Julia Valentim Tavares[1,2], Emanuel Gloor[2], Thiago S. F. Silva[3], Rafael S. Oliveira[4], Fernanda Coelho de Souza[5], Caroline Signori-Müller[6,4], Francisco Carvalho Diniz[2], Luciano Pereira[7], Martin Acosta[8], Martin Gilpin[2], Manuel J. Marca Zevallos[9,10], Carlos A. Salas Yupayccana[9], Flor M. Perez-Mullisaca[9], Halina Jancoski[11], Marina Corrêa Scalon[12], Beatriz Schwantes Marimon[11], Ben Hur Marimon Junior[11], Yadvinder Malhi[13], Imma Oliveras Menor[14,13], Lucy Rowland[6], Patrick Meir[15,16], Paulo Bittencourt[17], Antonio Carlos Lola da Costa[18,19], João Antônio R. Santos[20], Renata Teixeira de Oliveira[20] , Adriane Esquivel-Muelbert[21,22], Esteban Álvarez-Dávila[23], Miguel N. Alexiades[24], Edmar Almeida de Oliveira[11], Ana Andrade[25], Luiz Aragão[26], Alejandro Araujo-Murakami[27,28], Luzmila Arroyo[28], Gerardo Aymard[29], Jorcely G. Barroso[30], Damien Bonal[31], Roel Brienen[2], Carlos Céron[32], José Luís Camargo[25], Richarlly Silva[33], Wendeson Castro[20], Jérôme Chave[34], James Comiskey[35,36], Douglas C Daly[37], Geraldine Derroire[38,39,40], Mathias Disney[41], Aurelie Dourdain[38], Sophie Fauset[42], Ted Feldpausch[6], Gerardo Flores Llampazo[43,], Bruno Hérault[39,44], Lionel Hernández[45], Niro Higuchi[46], Eurídice N. Honorio Coronado[47], Eliana Jimenez-Rojas[48], Michelle Kalamandeen[49], Susan Laurance[50], William Laurance[50], Simon Lewis[2,41], Antonio S Lima[19], Abel Monteagudo-Mendoza[9,51], Paulo Morandi[11], Percy Núñez Vargas[9], David Neill[52 (Deceased)], Walter Palacios[53], Alexander Parada Gutierrez[29], Guido Pardo-Molina[54], Maria Cristina Peñuela-Mora[55], Nigel Pitman[56], Rocio Rojas[51], Adriana Prieto[57], Maxime Réjou-Méchain[14], Hirma Ramírez-Angulo[58], Sabina Cerruto Ribeiro[59], Kalle Ruokolainen[60,61], Rafael P. Salomão[19,62], Julio Serrano[46], Rodrigo Sierra[63], Ademir R. Ruschel[64], Marcos Silveira[20], Hans ter Steege[65,66], John Terborgh[67,68], Luis Valenzuela Gamarra[51], Rodolfo Vásquez Martinez[51], Ima Vieira[19], Emilio Vilanova Torre[69], Vincent A. Vos[54], Ophelia Wang[70], Kenneth Young[70,71], Robert Muscarella[1], Kyle G. Dexter[15,73], Timothy R. Baker[2], Oliver L. Phillips[2], Maurizio Mencuccini[74,75], David Galbraith[2]

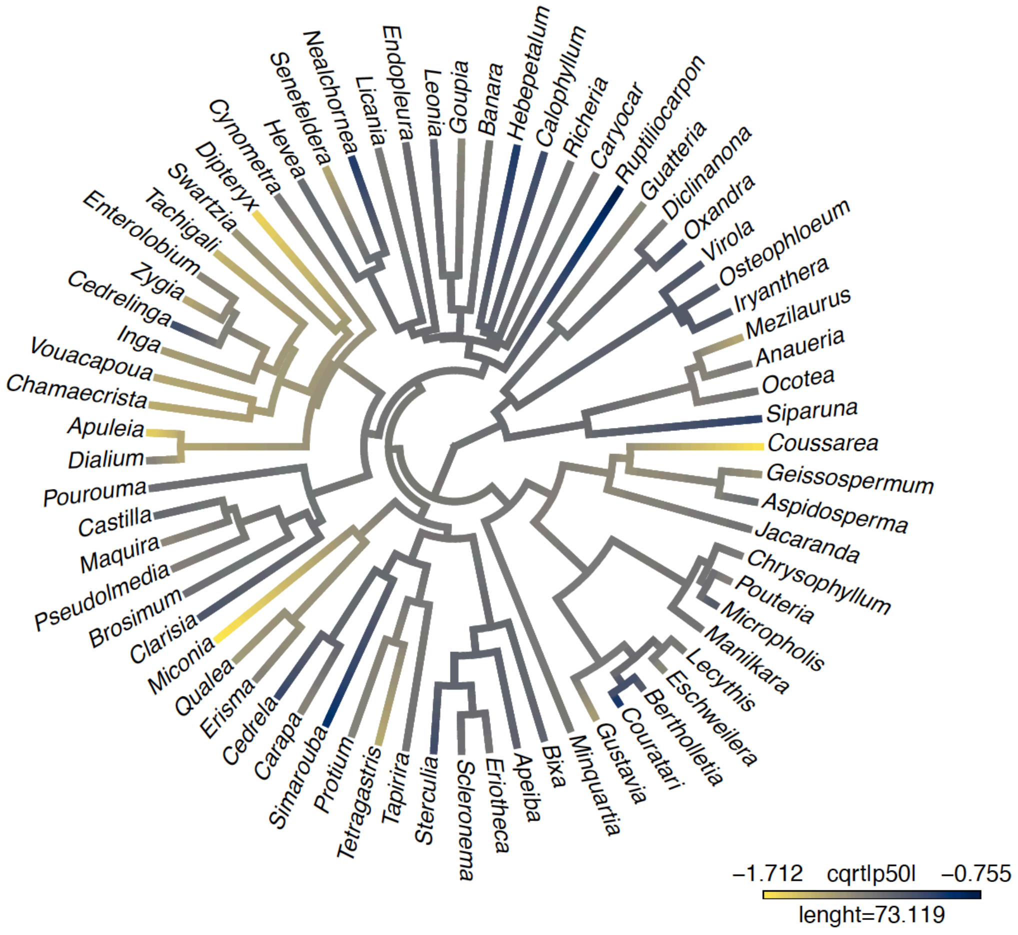

SI Figure 1. Phylogenetic tree[1] of Amazonian tree genera accounting for environmental variation by excluding forests with long dry season length in the dry fringe of Amazonia, n= 67 genera. Branches are coloured as transformed absolute mean Ψ_{50} values per genus.

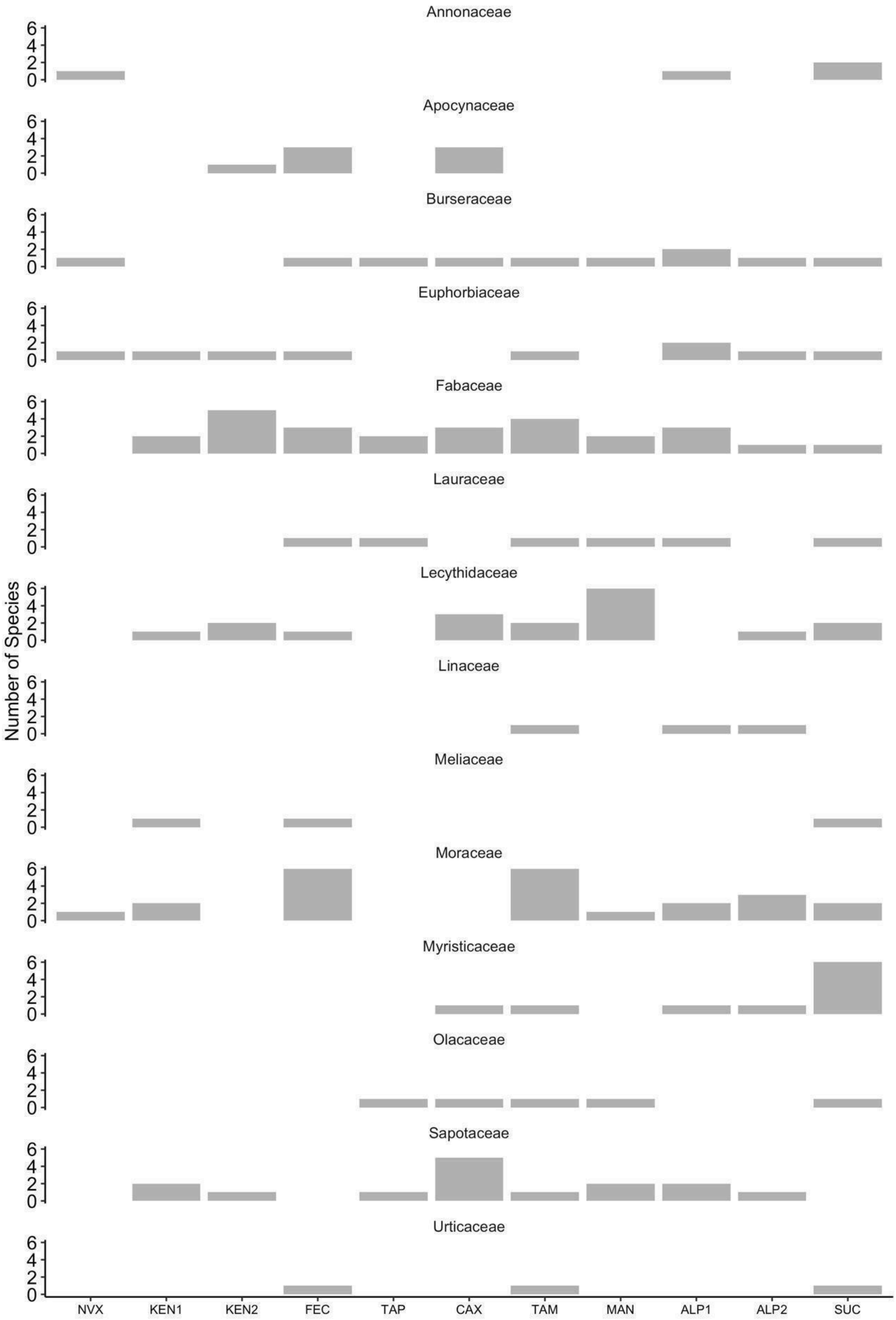

SI Figure 2. Distribution of widely occurring families across sites, encompassing only families that occurred in at least 3 sites along a wide climatological gradient. Sites from ref. [2,3] are ordered left to right as drier (NVX) to wetter (SUC) environments: long dry season

length - DSL (NVX, KEN1, KEN2); intermediate DSL (FEC, TAP, CAX, TAM, MAN) and ever-wet aseasonal forests (ALP1, ALP2, SUC).

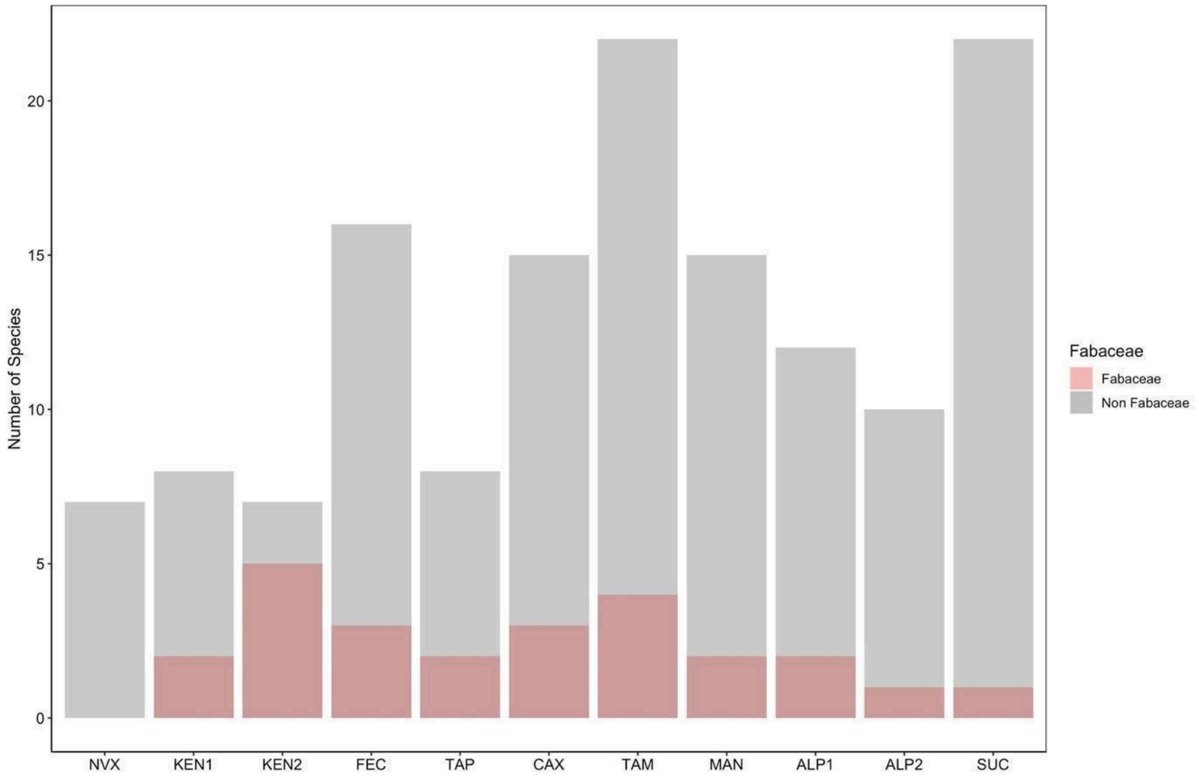

SI Figure 3. Distribution of Fabaceae and Non Fabaceae across the pan-Amazonian hydraulic traits dataset [2,3] (please see methods). Sites are ordered left to right as drier (NVX) to wetter (SUC) environments: long dry season length - DSL (NVX, KEN1, KEN2); intermediate DSL (FEC, TAP, CAX, TAM, MAN) and ever-wet aseasonal forests (ALP1, ALP2, SUC).

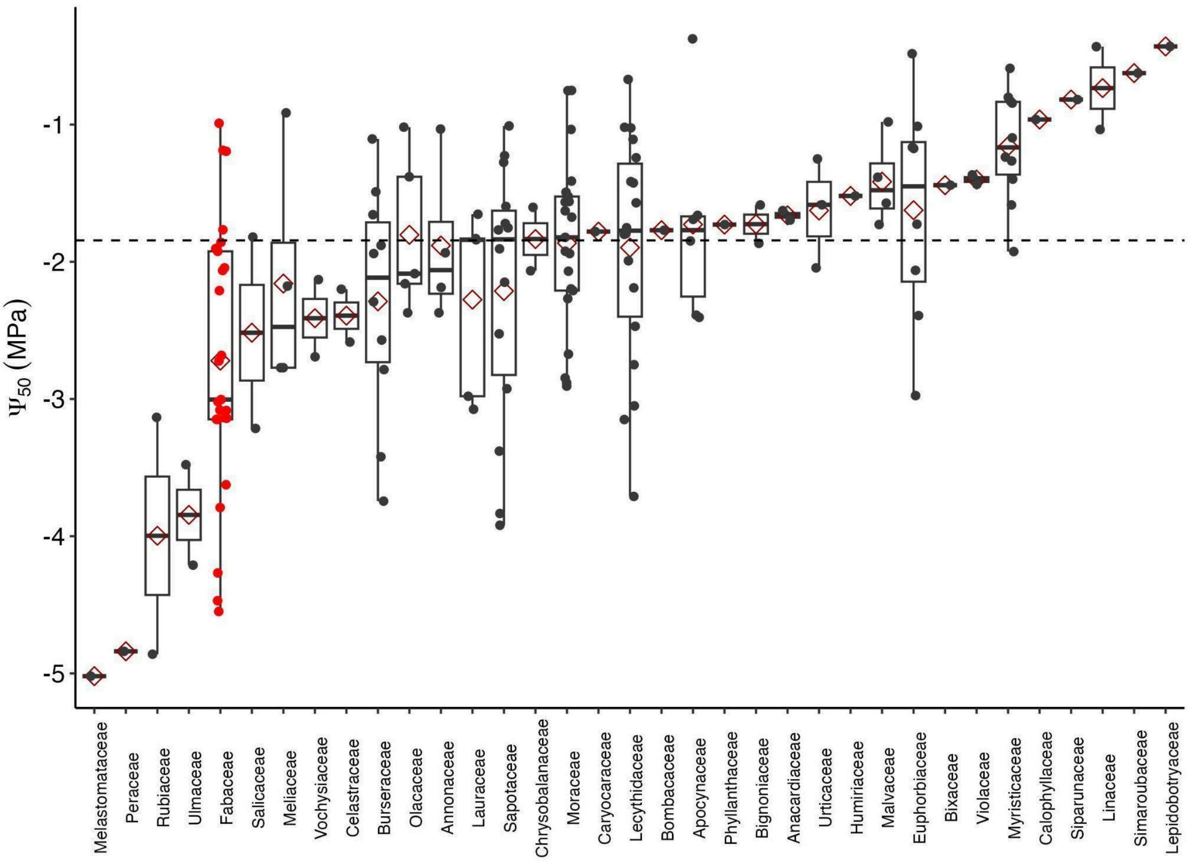

SI Figure 4. Embolism resistance variation across all sampled families in the dataset, regardless of sampling density across site and species. Dashed horizontal lines show mean trait value across families. Boxplots show the 25th percentile, median and 75th percentile. Vertical bars show the interquartile range ×1.5 and data points beyond these bars are potential outliers. The dashed vertical line shows the mean Ψ_{50} across the pan-Amazonian dataset[2,3] .

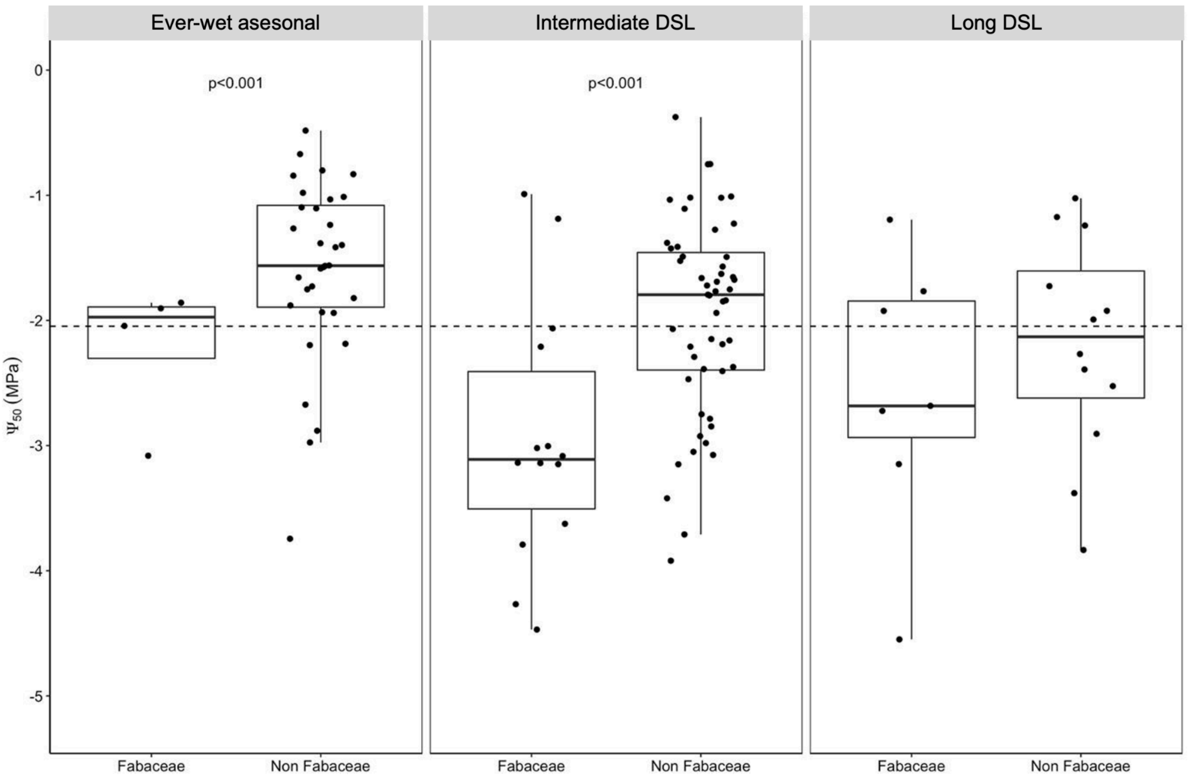

SI Figure 5. Difference in embolism resistance (Ψ_{50}) between Fabaceae and non-Fabaceae across Amazonian forest types: ever-wet asesonal forest, forests with intermediate dry season length - DSL and long DSL, considering the entire dataset [2,3] (please see methods). Statistical differences at $p<0.05$ using Wilcoxon rank sum tests are displayed on the figure.

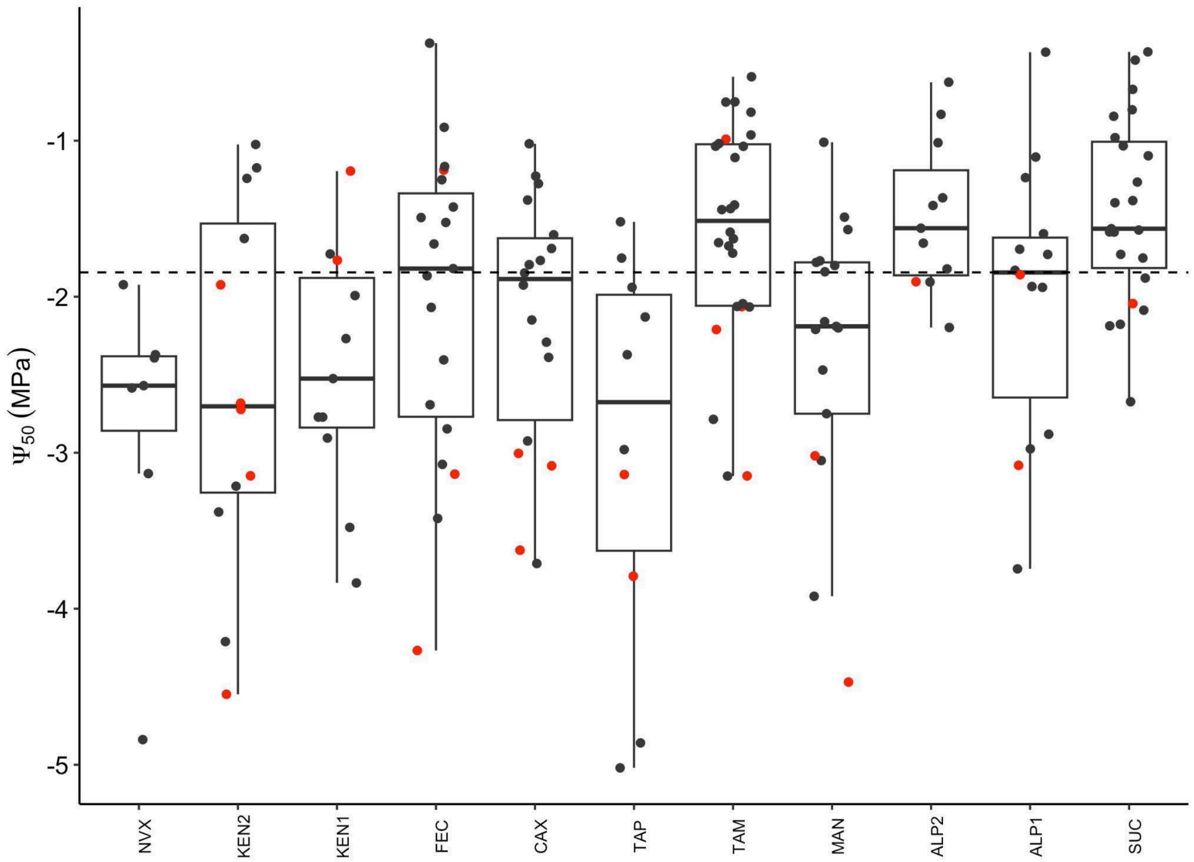

SI Figure 6. Embolism resistance variation across sites [2,3], which are are ordered left to right as drier (NVX) to wetter (SUC) environments: long dry season length - DSL (NVX, KEN1, KEN2); intermediate DSL (FEC, TAP, CAX, TAM, MAN) and ever-wet aseasonal forests (ALP1, ALP2, SUC). Boxplots show the 25th percentile, median and 75th percentile. Vertical bars show the interquartile range ×1.5 and data points beyond these bars are potential outliers. The dashed horizontal line shows the mean Ψ_{50} across the pan-Amazonian dataset. Red points represent Fabaceae species.

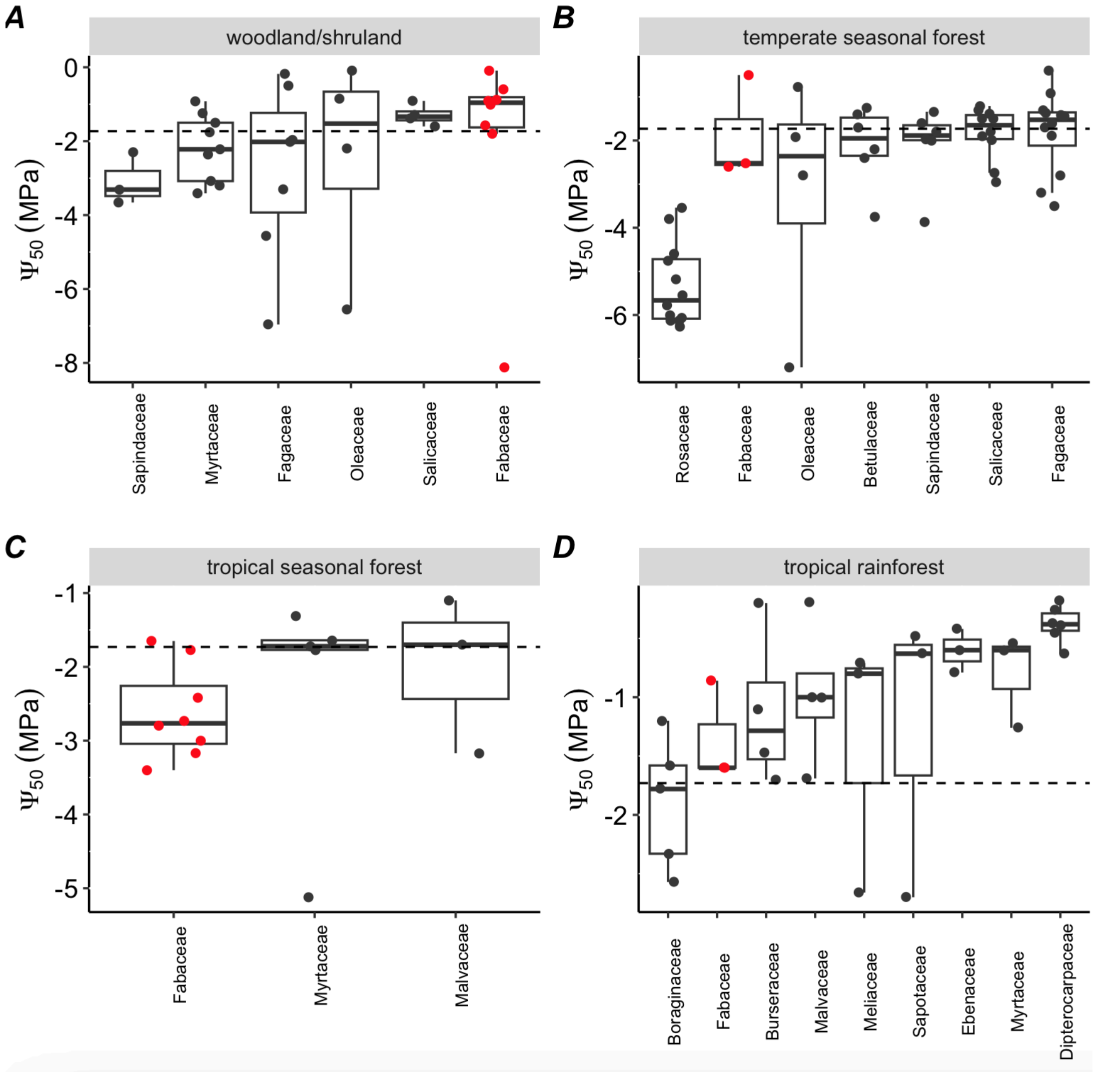

SI Figure 7. Xylem embolism resistance (Ψ_{50}) across angiosperm families in different biomes, based on the global dataset from ref. [4]. Panels show data for: (A) woodland/shrubland, (B) temperate seasonal forest, (C) tropical seasonal forest, and (D) tropical rainforest. Red points indicate Fabaceae species; black points represent species of other families. Dashed lines represent biome-wide means.

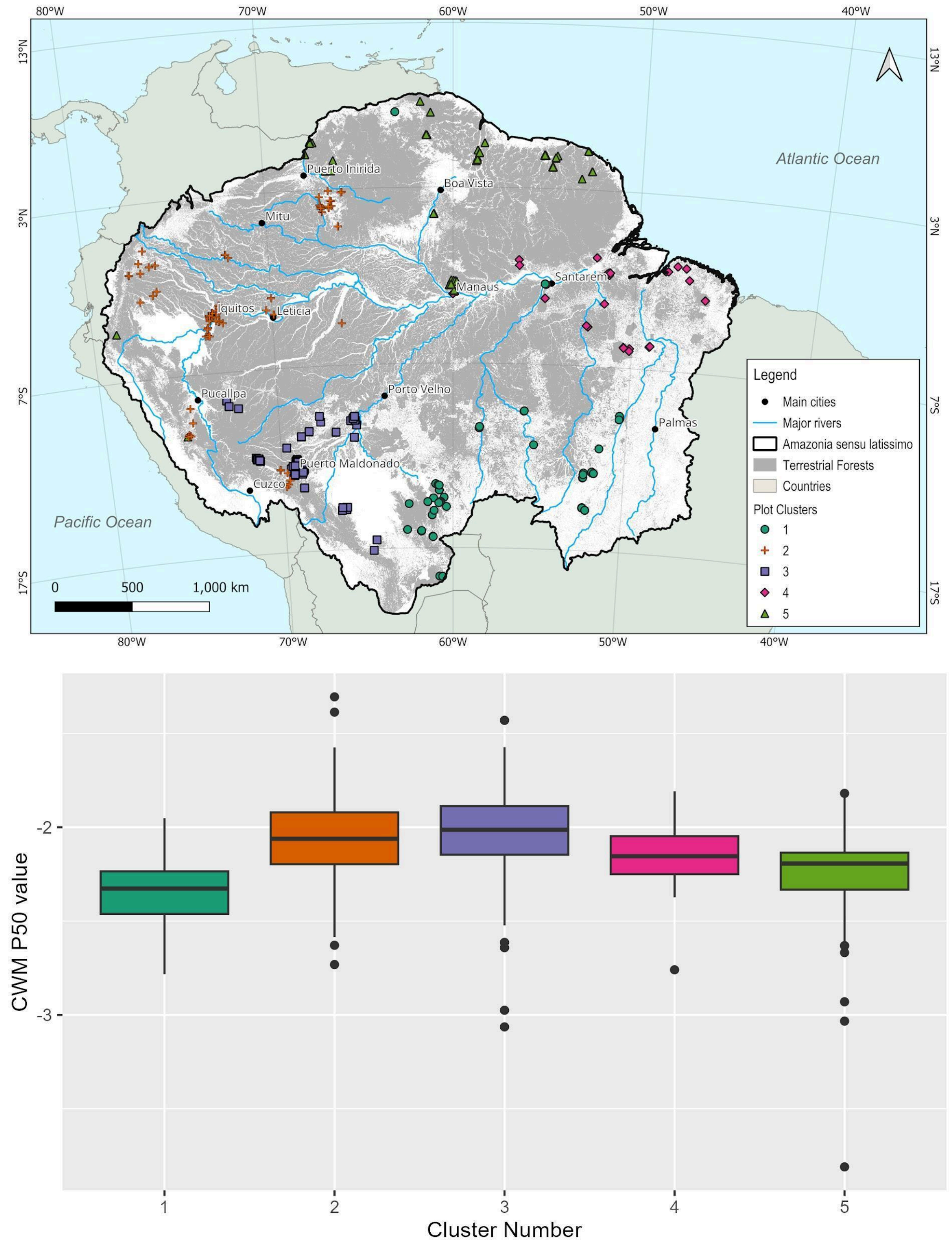

SI Figure 8. Top: results of the geographically constrained clustering of plots based on the combination of Ψ_{50}, MCWD (Mean Cumulative Water Deficit) and WTD (Water Table Depth) for the 448 inventoried non-flooded upland moist forest plots distributed across Amazonia *sensu latissimo* (see methods). Areas with very different edaphic environments - flooded forests, white sand forests and deforested areas – are masked from the background (see methods). Bottom: Distribution of Ψ_{50} values within each spatial cluster.

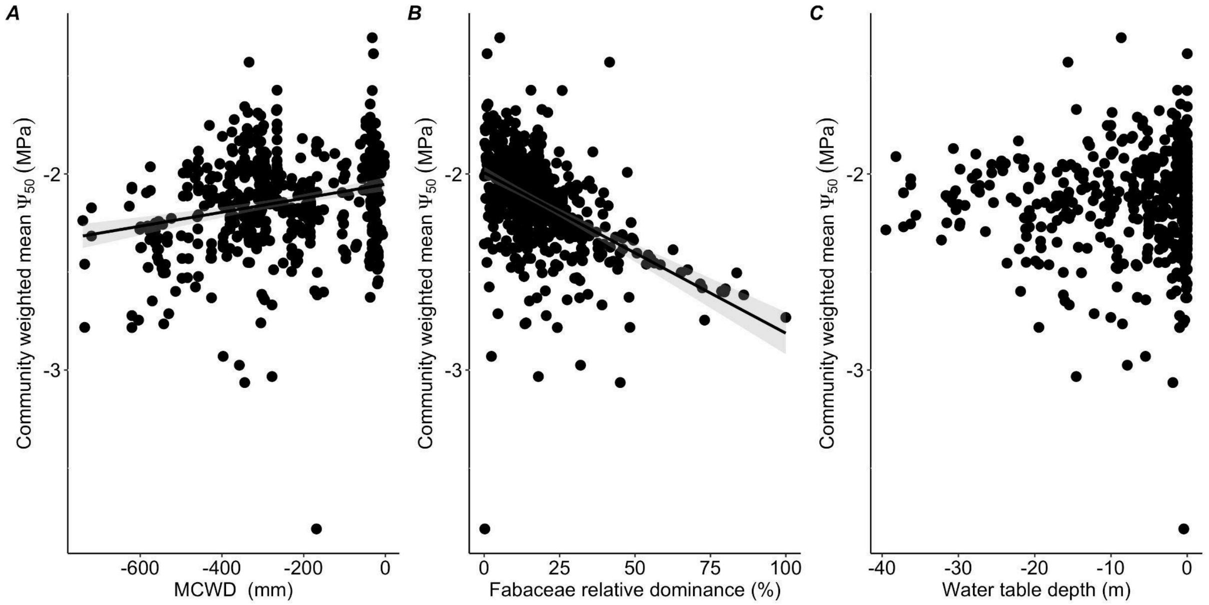

SI Figure 9. Estimated community-weighted mean embolism resistance relationships with (A) Maximum Climatological Water Deficit, (B) Fabaceae Relative Dominance per plot, (C) Water Table Depth. Black points represent 448 permanent *terra-firme* plots across Amazonia. Significant linear relations are shown by regression lines, 95% confidence intervals by shaded areas on the figure. Statistical results panel A: RSE=0.24, R^2=0.0315; F=15.38, df=441, p=0.0001; panel B: RSE=0.2141, R^2=0.2426; F=143.9, df=445, $p<2.2e^{-16}$; panel C: RSE=0.246, R^2=0.002786; F=1.61, DF=446, p=0.2052; Information about water table depth was obtained from a raster file available at ref [5].

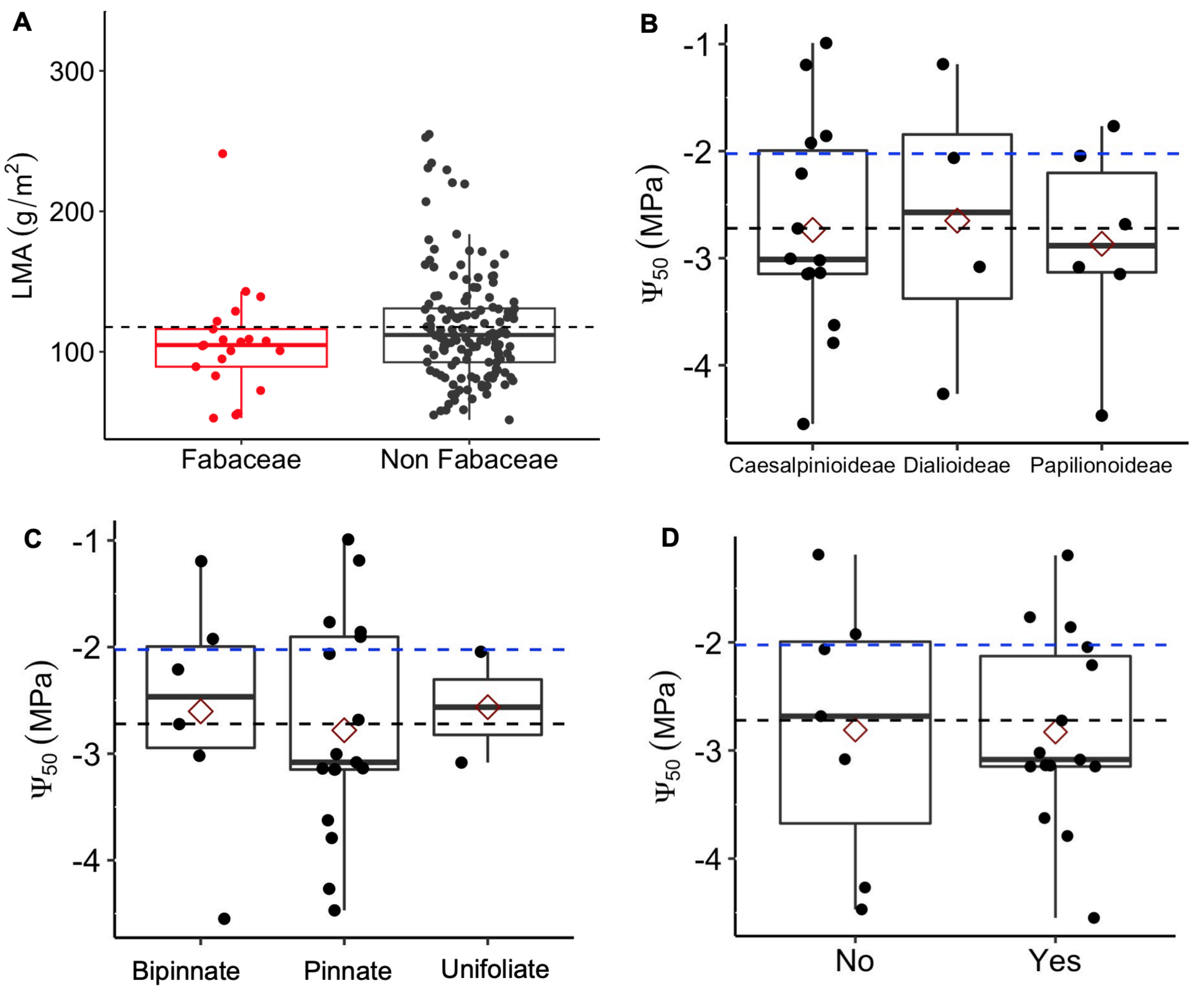

SI Figure 10. Fabaceae embolism resistance in variation across leaf traits, phylogenetic subgroups and nitrogen fixation ability. (A) Comparison of leaf mass per area (LMA) between Fabaceae (red) and non-Fabaceae (black) species across our entire dataset. (B–D) Variation in xylem embolism resistance (Ψ_{50}) within Fabaceae subgroups: (B) by phylogenetic subfamily (Caesalpinioideae, Dialioideae, Papilionoideae), (C) by leaf architecture (bipinnate, pinnate, unifoliate), and (D) by nitrogen-fixation ability (yes/no; based on ref. [6]. Red diamonds indicate group means; black dashed lines indicate the overall Fabaceae mean Ψ_{50}; blue dashed lines represent the overall dataset mean.

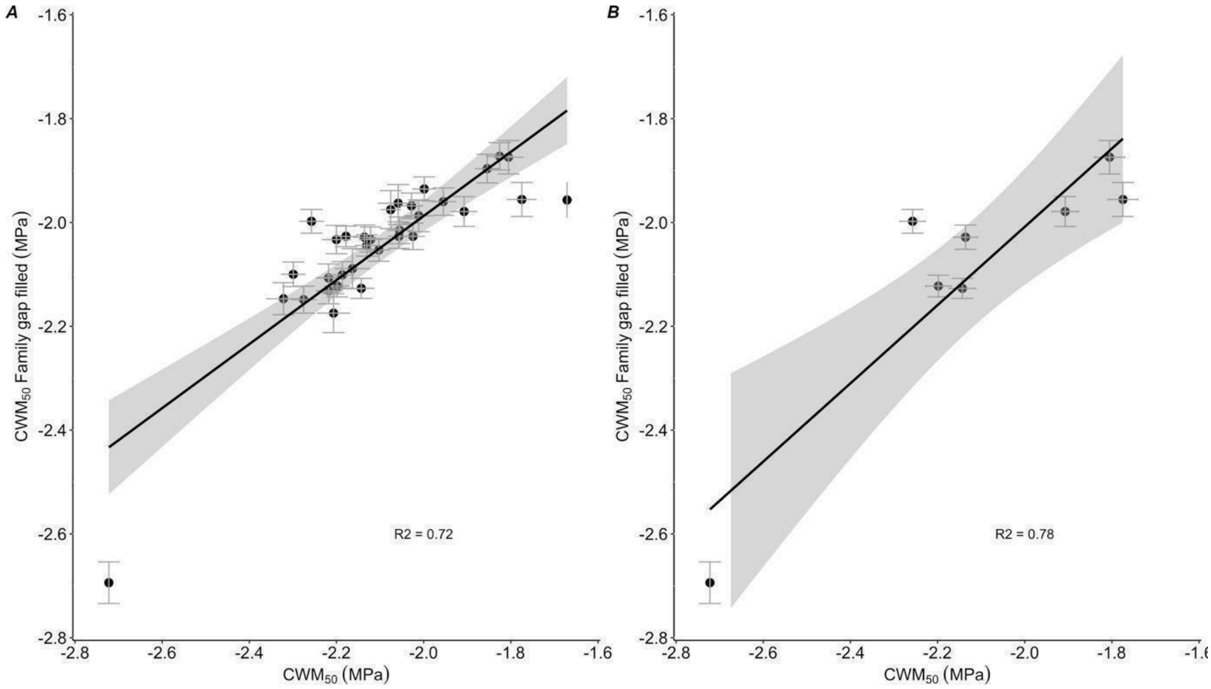

SI Figure 11. Gap filling test. To validate the gap-filling process (please see methods), we simulated the community weighted mean embolism resistance (CWM Ψ_{50}) by replacing original species values with family-mean values in plots with Ψ50 data[2,3] – B panel – and for the surrounding plots with similar species composition (A panel). Significant linear relations are shown by regression lines, 95% confidence intervals, by shaded areas and the R^2 of each regression is shown on the figure.

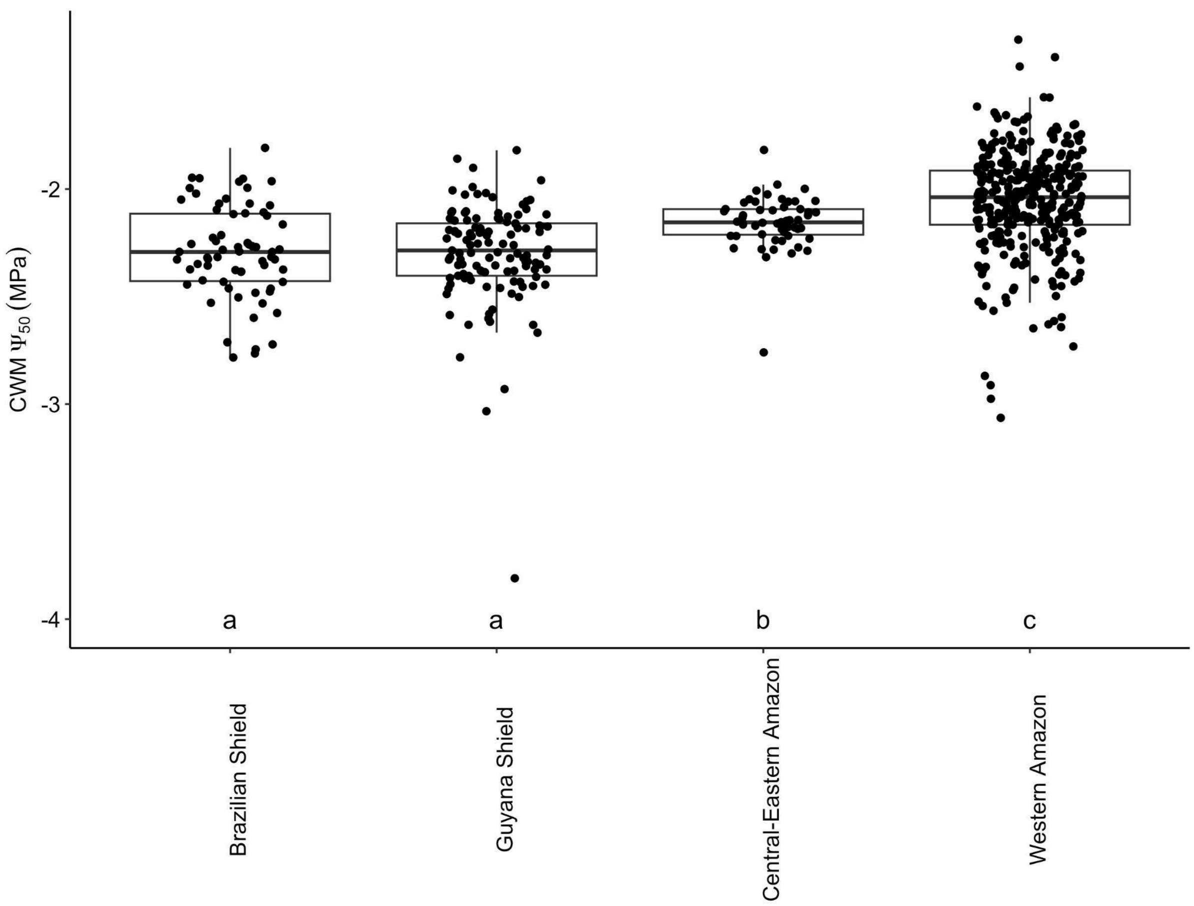

SI Figure 12. Estimated community-weighted mean embolism resistance of *terra-firme* 448 plots across Amazonian regions[7]. Differences at 0.05 level of significance using Pairwise Wilcoxon Rank Sum test are indicated by letters below each boxplot. Black points represent forest sites (n=448).

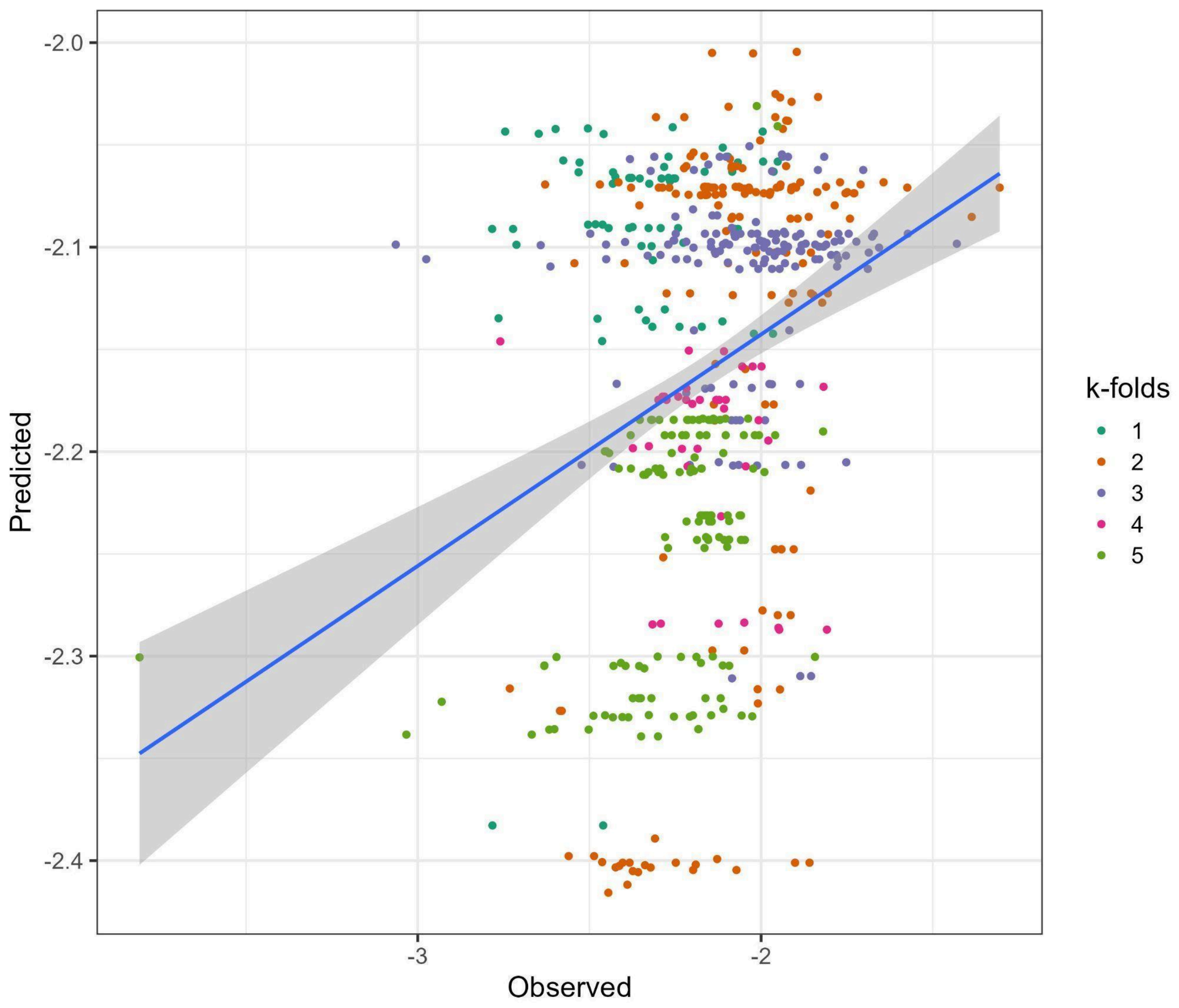

SI Figure 13. Observed versus interpolated (predicted) Ψ_{50} values based on five-fold cross-validation, showing how the smoothing and averaging that results from the IDW interpolation method used reduced the range of Ψ_{50} values to between -2 MPa and -2.4 MPa, versus -1.3 MPa to -3.8 MPa in the observed data, and explaining the low quantitative agreement at the local (point/pixel) level.

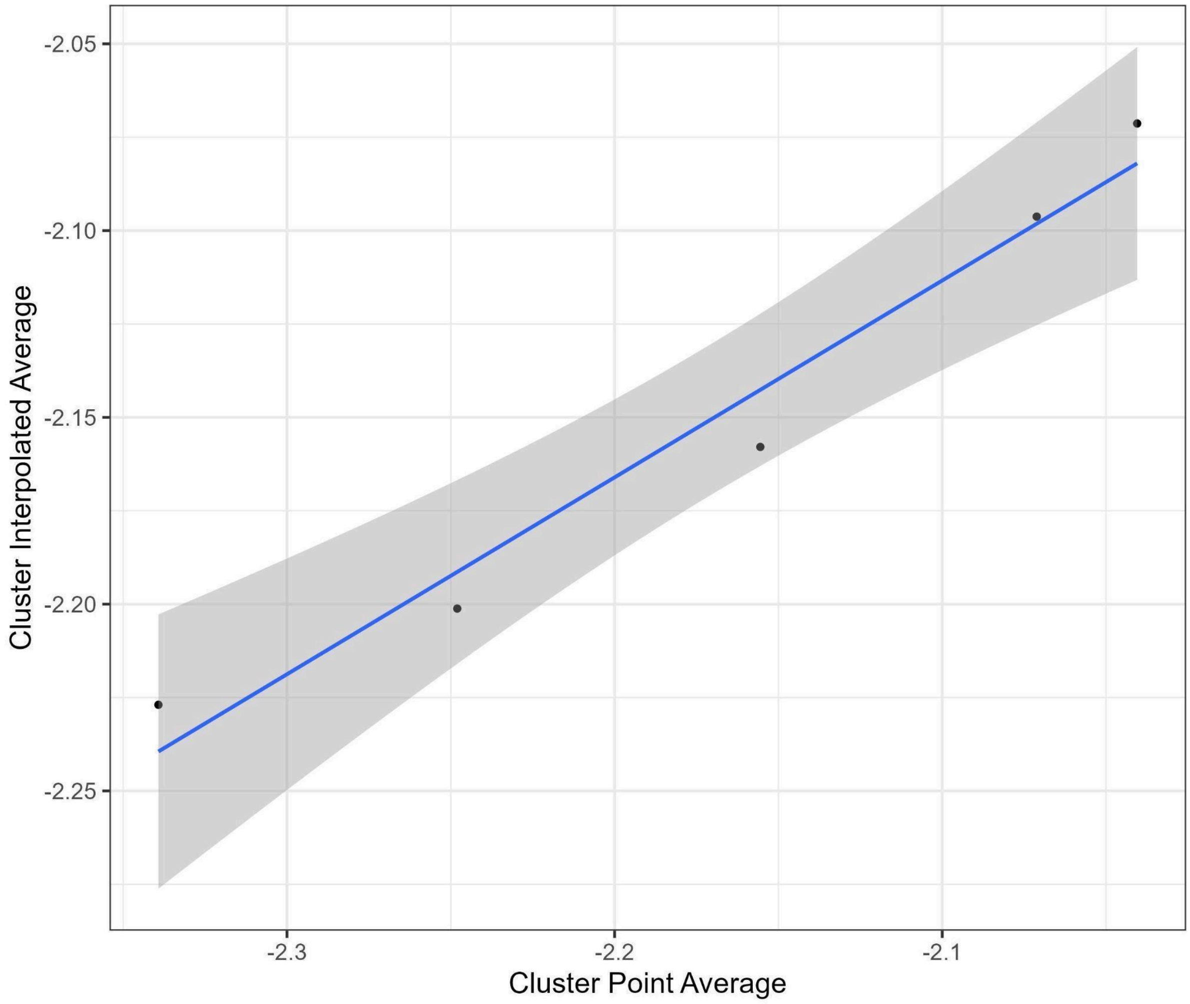

SI Figure 14. Agreement between average Ψ_{50} values calculated from the field-based estimates, for each spatial cluster (SI Figure 8), and average Ψ_{50} values calculated from interpolated data, for the area determined by the minimum convex hull enclosing each spatial cluster.

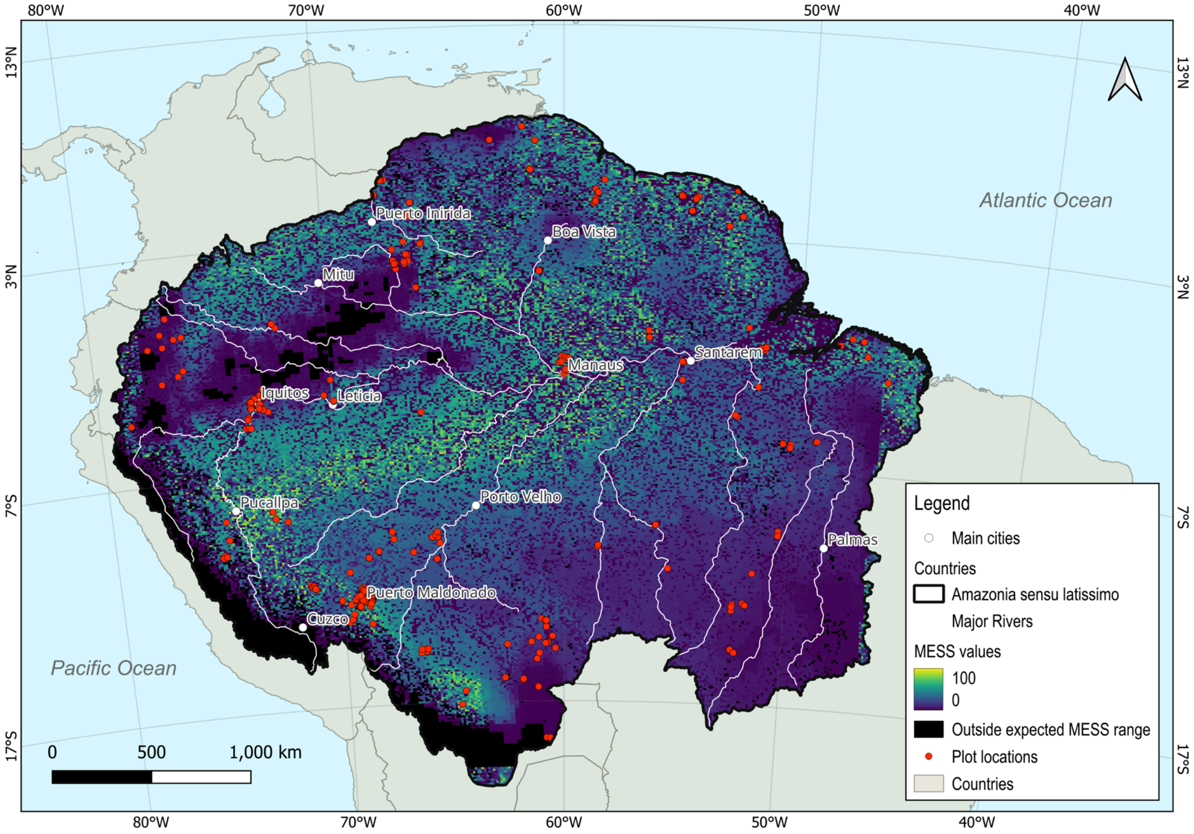

SI Figure 15. Multivariate Environmental Similarity Surfaces (MESS) analysis of the interpolated surface, using Maximum Cumulative Water Deficit and Water Table Depth layers as environmental variables, and their corresponding values for each of the 448 plots as the reference values for MESS calculation.

References

1. Coelho de Souza, F. *et al.* Evolutionary diversity is associated with wood productivity in Amazonian forests. *Nat. Ecol. Evol.* 3, 1754–1761 (2019).

2. Tavares, J. V. *et al.* Data and R-code from 'Julia Valentim Tavares, Rafael S. Oliveira, Maurizio Mencuccini, [...] Oliver L. Phillips, Emanuel Gloor, David R. Galbraith. Basin-wide variation in tree hydraulic safety margins predicts the carbon balance of Amazon forests. Nature 2023. DOI 10.1038/s41586-023-05971-3'. ForestPlots.net https://doi.org/10.5521/FORESTPLOTS.NET/2023_1 (2023).

3. Tavares, J. V. *et al.* Basin-wide variation in tree hydraulic safety margins predicts the carbon balance of Amazon forests. *Nature* 617, 111–117 (2023).

4. Choat, B. *et al.* Global convergence in the vulnerability of forests to drought. *Nature* 491, 752–755 (2012).
5. Fan, Y., Li, H. & Miguez-Macho, G. Global patterns of groundwater table depth. *Science* 339, 940–943 (2013).
6. Gei, M. *et al.* Legume abundance along successional and rainfall gradients in Neotropical forests. *Nat. Ecol. Evol.* 2, 1104–1111 (2018).
7. Feldpausch, T. R. *et al.* Height-diameter allometry of tropical forest trees. *Biogeosciences* 8, 1081–1106 (2011).
