# Supplementary material for: Family imprint reveals basin-wide patterns of Amazon forest embolism resistance
Source: Nat Commun. 2026 Feb 26;17:2073. doi: 10.1038/s41467-026-69892-1 (PMC12949053; doi:10.1038/s41467-026-69892-1)
Supplement: Supplementary file 2 — Reporting Summary [file 41467_2026_69892_MOESM2_ESM.pdf]

nature portfolio

Corresponding author(s): Julia Valentim Tavares
Last updated by author(s): Dec 2, 2025

Reporting Summary

Nature Portfolio wishes to improve the reproducibility of the work that we publish. This form provides structure for consistency and transparency in reporting. For further information on Nature Portfolio policies, see our Editorial Policies and the Editorial Policy Checklist.

Statistics

For all statistical analyses, confirm that the following items are present in the figure legend, table legend, main text, or Methods section.

n/a	Confirmed	
☐	☒	The exact sample size (*n*) for each experimental group/condition, given as a discrete number and unit of measurement
☐	☒	A statement on whether measurements were taken from distinct samples or whether the same sample was measured repeatedly
☐	☒	The statistical test(s) used AND whether they are one- or two-sided<br>*Only common tests should be described solely by name; describe more complex techniques in the Methods section.*
☐	☒	A description of all covariates tested
☐	☒	A description of any assumptions or corrections, such as tests of normality and adjustment for multiple comparisons
☐	☒	A full description of the statistical parameters including central tendency (e.g. means) or other basic estimates (e.g. regression coefficient) AND variation (e.g. standard deviation) or associated estimates of uncertainty (e.g. confidence intervals)
☐	☒	For null hypothesis testing, the test statistic (e.g. *F*, *t*, *r*) with confidence intervals, effect sizes, degrees of freedom and *P* value noted<br>*Give P values as exact values whenever suitable.*
☒	☐	For Bayesian analysis, information on the choice of priors and Markov chain Monte Carlo settings
☒	☐	For hierarchical and complex designs, identification of the appropriate level for tests and full reporting of outcomes
☒	☐	Estimates of effect sizes (e.g. Cohen's *d*, Pearson's *r*), indicating how they were calculated

Our web collection on statistics for biologists contains articles on many of the points above.

Software and code

Policy information about availability of computer code

Data collection

No software was used for data collection for this study. All inventory forest data were uploaded and are curated at ForestPlots.net (ForestPlots.net et al., 2021). Phylogenetic tree was obtained from Coelho de Sousa et al., 2018. Tree embolism resistance data were obtained from the pan-Amazonian hydraulic trait dataset (Tavares et al., 2023), which encompasses previously published data from Central-Eastern Amazon (Brum et al., 2018, Barros et al., 2019, Bittencourt et al., 2020) and newly collected data from Western and Southern Amazon (Tavares et al., 2023).

References:
ForestPlots.net et al. (2021) 'Taking the pulse of Earth ' s tropical forests using networks of highly', 260(September 2020).
Coelho de Souza, F. et al. (2016) 'Evolutionary heritage influences amazon tree ecology', Proceedings of the Royal Society B: Biological Sciences, 283(1844).
Tavares, J. V. et al. (2023) 'Basin-wide variation in tree hydraulic safety margins predicts the carbon balance of Amazon forests', Nature, 617(May).
Brum, M. et al. (2018) 'Hydrological niche segregation defines forest structure and drought tolerance strategies in a seasonal Amazon forest', Journal of Ecology, (January),
Barros, F. de V. et al. (2019) 'Hydraulic traits explain differential responses of Amazonian forests to the 2015 El Niño-induced drought', New Phytologist, pp. 1253–1266.
Bittencourt, P. R. L. et al. Amazonia trees have limited capacity to acclimate plant hydraulic properties in response to long-term drought. Glob. Change Biol. 26, 3569–3584 (2020).

Data analysis

All analyses were performed using the open source R version 4.4.0 (2024-04-24) -- "Puppy Cup" - Copyright (C) 2024 The R Foundation for

Data analysis | Statistical Computing

For manuscripts utilizing custom algorithms or software that are central to the research but not yet described in published literature, software must be made available to editors and reviewers. We strongly encourage code deposition in a community repository (e.g. GitHub). See the Nature Portfolio guidelines for submitting code & software for further information.

Data

Policy information about availability of data

All manuscripts must include a data availability statement. This statement should provide the following information, where applicable:

- Accession codes, unique identifiers, or web links for publicly available datasets
- A description of any restrictions on data availability
- For clinical datasets or third party data, please ensure that the statement adheres to our policy

Data and codes to recreate the main analyses and the main figures presented in this study will be available as a ForestPlots.net data package associated with this study: https://doi.org/10.5521/forestplots.net/2025_5.

Research involving human participants, their data, or biological material

Policy information about studies with human participants or human data. See also policy information about sex, gender (identity/presentation), and sexual orientation and race, ethnicity and racism.

Reporting on sex and gender: This study does not involve human participants, their data or biological material.

Reporting on race, ethnicity, or other socially relevant groupings: This study does not involve human participants, their data or biological material.

Population characteristics: This study does not involve human participants, their data or biological material.

Recruitment: This study does not involve human participants, their data or biological material.

Ethics oversight: This study does not involve human participants, their data or biological material.

Note that full information on the approval of the study protocol must also be provided in the manuscript.

Field-specific reporting

Please select the one below that is the best fit for your research. If you are not sure, read the appropriate sections before making your selection.

☐ Life sciences ☐ Behavioural & social sciences ☒ Ecological, evolutionary & environmental sciences

For a reference copy of the document with all sections, see nature.com/documents/nr-reporting-summary-flat.pdf

Ecological, evolutionary & environmental sciences study design

All studies must disclose on these points even when the disclosure is negative.

Study description: Our study leverages extensive forest inventory data and phylogenetic analysis to address a major challenge in understanding Amazonian forest resistance to drought at a very large scale. Here we produced produce a comprehensive macroecological assessment of Amazonian vulnerability to embolism, an essential metric of tree ability to persist under dry conditions, by using data from the largest tree hydraulic traits dataset assessed to date (Tavares et al., 2023) combined with 448 terrestrial forest inventory plots over the entire Amazon basin (ForestPlots.net et al., 2021).

Reference:
ForestPlots.net et al. (2021) 'Taking the pulse of Earth ' s tropical forests using networks of highly', 260(September 2020).
Tavares, J. V. et al. (2023) 'Basin-wide variation in tree hydraulic safety margins predicts the carbon balance of Amazon forests', Nature, 617(May).

Research sample: To assess geographic patterns of embolism resistance across the Amazon, we combined the Ψ_{50} phylogenetic signal detected across Amazonian trees with inventory data to computed community-weighted mean Ψ_{50} values for 448 structurally mature lowland tropical forest plots distributed across Amazonia. Tree-level data were obtained from the RAINFOR inventory network via the ForestPlots.net database (ForestPlots.net et al., 2021). For each plot, we selected a single census with the highest available level of taxonomic identification at the species level. We restricted our selection to plots located within Amazonia sensu latissimo (Eva et al., 2005), excluding dry forests, flooded forests, plots situated above 1000 m in elevation, and plots affected by direct human disturbance. This approach ensured that analyses focused on intact terra-firme (non-flooded) forests representative of the major Amazonian biogeographic regions.

Reference:

ForestPlots.net et al. (2021) 'Taking the pulse of Earth ' s tropical forests using networks of highly', 260(September 2020).
Eva, H. D. et al., (2005). A proposal for defining the geographical boundaries of Amazonia; synthesis of the results from an expert consultation workshop organized by the European Commission in collaboration with the Amazon Cooperation Treaty Organization-JRC Ispra, 7-8 June 2005 (No. 21808-EN). European Commission.

Sampling strategy

We compiled a pan-Amazonian dataset of embolism resistance (Ψ50) from 129 tree species across 88 genera and 36 families, sampled across 11 sites representing the Amazon's climatological gradient, including forests with long, intermediate, and no dry season. Species-level Ψ50 values were derived from xylem vulnerability curves using pneumatic and bench dehydration methods. For macroecological analyses, we combined these data with plot-level species composition from 448 RAINFOR inventory plots across lowland undisturbed Amazonian forests. To ensure robust coverage and taxonomic consistency, we restricted analysis to plots sharing at least 60% of basal area family composition with the Ψ50 dataset. Missing trait data were gap-filled hierarchically (genus, family, plot mean), and community-weighted means were computed based on species relative dominance. To visualize macroecological patterns, we applied a geographically constrained clustering analysis and an optimized Inverse Distance Weighting (IDW) interpolation across the Amazon basin.

Data collection

Hydraulic traits were collect as defined in Tavares et al. (2023)
Forest inventory data were originated from the RAINFOR network, which coordinates standardized forest inventory plots across the Amazon basin. Individual plots were established and monitored following standardized protocols to ensure consistency in tree measurement and taxonomic identification. Data used in this study were accessed through the ForestPlots.net database and were collected with appropriate permissions and collaboration agreements between participating institutions and local authorities. No new field data were collected specifically for this study.

Reference:
Tavares, J. V. et al. (2023) 'Basin-wide variation in tree hydraulic safety margins predicts the carbon balance of Amazon forests', Nature, 617(May).

Timing and spatial scale

Does not apply.

Data exclusions

Plots were excluded if they were located outside Amazonia sensu latissimo, situated above 1000 m elevation, classified as dry forests or seasonally/flooded forests, or if there was evidence of recent anthropogenic disturbance. Plots with insufficient species-level taxonomic identification were also excluded. Exclusion criteria were pre-defined before analysis to ensure consistency

Reproducibility

All details are described in the Methods session to all analyses presented in this study to be reproduced.

Randomization

We performed phylogenetic randomization analysis (Dexter and Chave, 2016) to investigate which specific taxonomic groups may strongly contribute to phylogenetic signal for embolism resistance of Amazonian trees.

Reference:
Dexter, K. and Chave, J. (2016) 'Evolutionary patterns of range size, abundance and species richness in Amazonian angiosperm trees', PeerJ, 4, p. e2402

Blinding

Blinding was not relevant to our study

Did the study involve field work? ☐ Yes ☒ No

Reporting for specific materials, systems and methods

We require information from authors about some types of materials, experimental systems and methods used in many studies. Here, indicate whether each material, system or method listed is relevant to your study. If you are not sure if a list item applies to your research, read the appropriate section before selecting a response.

Materials & experimental systems

n/a	Involved in the study
☒	☐ Antibodies
☒	☐ Eukaryotic cell lines
☒	☐ Palaeontology and archaeology
☒	☐ Animals and other organisms
☒	☐ Clinical data
☒	☐ Dual use research of concern
☐	☒ Plants

Methods

n/a	Involved in the study
☒	☐ ChIP-seq
☒	☐ Flow cytometry
☒	☐ MRI-based neuroimaging

Plants

Seed stocks	Seed stocks were not relevant to our study.
Novel plant genotypes	Does not apply
Authentication	Does not apply
